# Whole-Exome Sequencing Identifies Somatic Mutations Associated With Mortality in Metastatic Clear Cell Kidney Carcinoma

**DOI:** 10.3389/fgene.2019.00439

**Published:** 2019-05-15

**Authors:** Alejandro Mendoza-Alvarez, Beatriz Guillen-Guio, Adrian Baez-Ortega, Carolina Hernandez-Perez, Sita Lakhwani-Lakhwani, Maria-del-Carmen Maeso, Jose M. Lorenzo-Salazar, Manuel Morales, Carlos Flores

**Affiliations:** ^1^Research Unit, Hospital Universitario Nuestra Señora de Candelaria, Universidad de La Laguna, Santa Cruz de Tenerife, Spain; ^2^Department of Veterinary Medicine, University of Cambridge, Cambridge, United Kingdom; ^3^Service of Medical Oncology, Hospital Universitario Nuestra Señora de Candelaria, Santa Cruz de Tenerife, Spain; ^4^Department of Pathology, Hospital Universitario Nuestra Señora de Candelaria, Santa Cruz de Tenerife, Spain; ^5^Genomics Division, Instituto Tecnológico y de Energías Renovables, Santa Cruz de Tenerife, Spain; ^6^Instituto de Tecnologías Biomédicas, Universidad de La Laguna, Santa Cruz de Tenerife, Spain; ^7^Centro de Investigación Biomédica en Red de Enfermedades Respiratorias, Instituto de Salud Carlos III, Madrid, Spain

**Keywords:** ccRCC, whole-exome sequencing, kidney cancer, somatic mutation, mortality

## Abstract

Clear cell renal cell carcinoma (ccRCC) is among the most aggressive histologic subtypes of kidney cancer, representing about 3% of all human cancers. Patients at stage IV have nearly 60% of mortality in 2–3 years after diagnosis. To date, most ccRCC studies have used DNA microarrays and targeted sequencing of a small set of well-established, commonly altered genes. An exception is the large multi-omics study of The Cancer Genome Atlas Kidney Renal Clear Cell Carcinoma (TCGA-KIRC), which identified new ccRCC genes based on whole exome-sequencing (WES) data, and molecular prognostic signatures based on transcriptomics, epigenetics and proteomics data. Applying WES to simultaneously interrogate virtually all exons in the human genome for somatic variation, here we analyzed the burden of coding somatic mutations in metastatic ccRCC primary tumors, and its association with patient mortality from cancer, in patients who received VEGF receptor-targeting drugs as the first-line therapy. To this end, we sequenced the exomes of ten tumor–normal pairs of ccRCC patient tissues from primary biopsies at >100× mean depth and called somatic coding variation. Mutation burden analysis prioritized 138 genes linked to patient mortality. A gene set enrichment analysis evidenced strong statistical support for the abundance of genes involved in the development of kidney cancer (*p* = 2.31 × 10^−9^) and carcinoma (*p* = 1.22 × 10^−5^), with 49 genes having direct links with kidney cancer according to the published records. Two of these genes, *SIPA1L2* and *EIF3A*, demonstrated independent associations with mortality in TCGA-KIRC project data. Besides, three mutational signatures were found to be operative in the tumor exomes, one of which (COSMIC signature 12) has not been previously reported in ccRCC. Selection analysis yielded no detectable evidence of overall positive or negative selection, with the exome-wide number of nonsynonymous substitutions per synonymous site reflecting largely neutral tumor evolution. Despite the limited sample size, our results provide evidence for candidate genes where somatic mutation burden is tentatively associated with patient mortality in metastatic ccRCC, offering new potential pharmacological targets and a basis for further validation studies.

## Introduction

Clear cell renal cell carcinoma (ccRCC) represents only 2–3% of all human cancers (Manley et al., 2017). Notwithstanding, over 30% of ccRCC patients have metastases at the time of diagnosis, and 60% die in the first 2–3 years after diagnosis (Casuscelli et al., 2017). ccRCC is characterized by the resistance to radiation, cytotoxic and hormone therapies. Current treatments for ccRCC include diverse chemotherapeutic agents targeting the vascular endothelial growth factor (VEGF) pathway (Sternberg et al., 2010).

Roughly a decade ago, genetic approaches to disease diagnosis were postulated as a costly new way to progress toward the paradigm shift aimed by precision medicine. In ccRCC, the vast majority of studies have been directed at assessing genes that are known to be directly involved in pathogenesis, most of them using DNA arrays for genetic screening. The drawbacks and advantages of holistic vs. targeted gene studies have been extensively discussed in the literature (Iglesias et al., 2014; Kong et al., 2018). Nowadays, high-throughput next generation sequencing (NGS) technologies have made genetic testing affordable and cost-effective, hence consolidating as a central instrument for the progress toward the implementation of precision medicine. Furthermore, the reduction in per-base sequencing cost has popularized the use of whole-exome sequencing (WES) for the investigation of the pathogenic impact of genetic variation in coding regions (Damiati et al., 2016; Fay et al., 2016; Lata et al., 2018). This is reflected by the sheer number of WES studies being published, including a multitude of analyses of cancer exomes (Samarakoon et al., 2014; Lata et al., 2018).

To our knowledge, research in ccRCC using WES has previously focused on the treatment response or toxicity variables in relation to chemotherapeutic treatment. Moreover, kidney cancer studies were often limited to genes which are frequently altered in this condition, most commonly focusing on a gene panel conformed by *VHL*, *PBRM1*, *BAP1*, *SETD2*, *TP53*, *PTEN*, *KDM5C*, and *TERT* genes (Casuscelli et al., 2017; Manley et al., 2017; Tennenbaum et al., 2017). One notable exception to this is the study of more than 400 ccRCC patients with different omics approaches (The Cancer Genome Atlas Research Network, 2013). While this large study revealed more than 19 commonly mutated genes in ccRCC, molecular prognostic signatures were only pursued with transcriptomics, epigenetics, and proteomics data. Here, for the first time, we apply high-depth WES to assess the association between somatic mutation burden in metastatic ccRCC primary tumors, which refers to the total number of somatic mutations identified per gene per patient, and patient survival.

## Materials and Methods

### Patient Population and Setting

A total of 13 metastatic ccRCC patients (stage IV) from the two tertiary hospitals of Tenerife (Spain), Hospital Universitario Nuestra Señora de Candelaria (HUNSC) and Hospital Universitario de Canarias (HUC), were included in the study. The patients were all of European ancestry (self-declared), aged 31–80 years old (mean age of 56 years), with a male percentage of 61.5%. Seven (54%) of these patients died of cancer-related causes during the course of the study. The study was approved by the HUNSC Ethics Committee and written informed consent was obtained from all patients.

Nephrectomies were performed with curative intentions in six patients. For the rest of individuals, surgery was performed with cytoreductive purposes (Flanigan et al., 2001; Mickisch et al., 2001). Patients were classified into prognosis groups according to the Heng scoring system (Heng et al., 2013). At the moment of the diagnosis of metastasis, five patients showed good prognosis, while 6 had an intermediate prognosis and 2 a bad prognosis. All patients received tyrosine kinase inhibitors of the VEGF pathway, namely pazopanib (Sternberg et al., 2010) or sunitinib (Motzer et al., 2013), as the first-line treatment, except for one patient with bad prognosis who received temsirolimus (Hudes et al., 2007) as first-line treatment and pazopanib as second-line treatment.

Formalin-fixed paraffin-embedded (FFPE) biopsies from the primary tumors were obtained in blocks for subsequent DNA extraction. After evaluation by a pathologist, hematoxylin-eosin stained tissues were used to determine the limits of tumoral tissues. Whenever possible, nontumoral (thereafter referred to as normal) and tumoral tissues for DNA isolation were obtained from independent tissue slices. The GeneRead DNA FFPE Kit (QIAGEN, Hilden, Germany) was used for DNA isolation according to manufacturer’s instructions. The integrity and concentration of DNA was evaluated with the Qubit^®^ 3.0 Fluorometer, using the dsDNA BR Assay Kit (Thermo Fisher Scientific, Waltham, MA, United States), and the TaqMan^TM^ RNase P Detection Reagents Kit (Thermo Fisher Scientific, Waltham, MA, United States).

### Whole-Exome Sequencing

Enrichment, sequencing and read-mapping was accomplished by Macrogen Inc. Briefly, genomic DNA was enriched for exome regions using the Ion AmpliSeq^TM^ Exome RDY Kit (Thermo Fisher Scientific, Waltham, MA, United States) and Ion PI^TM^ Chip Kit v3 (Thermo Fisher Scientific, Waltham, MA, United States). Exome-enriched DNA was sequenced on the Ion Proton^TM^ platform (Thermo Fisher Scientific, Waltham, MA, United States), with two exomes per run to attain a theoretical depth of 100× per sample. Sequence data were aligned to the hg19/GRCh37 human reference genome using the Torrent Mapping Alignment Program v.5.0.13 included in the Torrent Suite Software for Sequencing Data Analysis v.5.0.4 (Thermo Fisher Scientific, Waltham, MA, United States).

### Variant Calling and Annotation

Aligned sequence data were analyzed to identify somatic and germline single-nucleotide variants (SNVs) and small insertions and deletions (indels). We called genetic variation using a computational pipeline built on the variant caller Platypus v0.8.1 (Rimmer et al., 2014) (Supplementary Figure S1). As part of the pipeline and based on our previous experience, Platypus was run twice on each BAM file with two different settings: (i) default mode with additional options –minReads = 3 and –minPosterior = 0, (ii) default mode with options –minReads = 3, –minPosterior = 0, –minFlank = 0 and –trimReadFlank = 10. Variants (SNVs and indels) flagged with Platypus quality flags “badReads,” “MQ,” “strandBias,” “SC,” and “QD” were subsequently discarded, and the remaining variants were merged into a single file and genotyped across each sample. Variants that continued to be flagged with “badReads,” “MQ,” “strandBias,” “SC,” and “QD” after this genotyping were discarded. These procedures (which include more stringent filters for SNVs called near indels) and the low read support threshold allowed achieving very high sensitivity in the initial calling, while the stages of filtering, genotyping and re-filtering of the variant calls ensured high specificity. We then filtered germline variation and retained somatic variants for subsequent analyses. To that aim, we filtered out the variants that were present in any of the normal tissues, as well as the variants that were supported by less than 3 sequence reads. Remaining variants were considered putative somatic variation and annotated using the Ensembl Variant Effect Predictor (VEP) v91.0 (McLaren et al., 2016), particularly for gene elements, coding consequences and deleteriousness according to SIFT scoring. In order to introduce a germline variation reference for comparisons in annotations, we accessed and annotated all common (>5%) variation described in the Exome Aggregation Consortium (ExAC) database, which incorporates 60,706 sequencing data of unrelated individuals. Finally, we evaluated the overlap of the putative somatic dataset with the data deposited in COSMIC (v87, released 13-Nov-18)^1^.

### Mutation Burden and Selection Analysis

We analyzed the annotated somatic variants in each gene using bespoke analysis routines coded in the R programming language (R Development Core Team, 2008). To rank the associations between the mutation burden and patient mortality, a Fisher’s exact test on the mutation count data was performed in R. A formal survival analysis adjusting for clinical and demographic confounders was not pursued because the minimal number of events that are needed per variable (Vittinghoff and McCulloch, 2007; Riley et al., 2018) was out of reach because of the limited sample size. However, with the exception of the treatment response, patients differing by mortality were not different for the major demographic and clinical variables (Table 1). Results were evaluated for inflation with a quantile-quantile (QQ) plot, using the qqplot v3.4.2 R package (Becker et al., 1988), and by estimating lambda with GenABEL v1.8-0 (Aulchenko et al., 2007). To assess evidence of positive or negative selection on somatic substitutions and detect any potential germline contamination in the somatic variant set, the dNdScv v0.0.0.9 R package (Martincorena et al., 2017) was employed to estimate exome-wide and per-gene number ratios of nonsynonymous substitutions per synonymous site (dN/dS).

**Table 1:** Demographic and clinical features of the study sample.

	Controls (*n* = 6)	Cases (*n* = 4)	*p*-value^∗^
Gender (% male)	67	75	1.00
Mean age (years)	56	58	0.61
Hypertension (%)	33	25	1.00
Smokers (%)	50	50	1.00
Drug toxicity (%)	33	50	1.00
Treatment response (% refractory)	17	100	0.05

*^∗^The age comparison was conducted by the Wilcoxon signed-rank test; the other variables were compared by a chi-square test. P25, percentile 25; P75, percentile 75.*

### Mutational Signature Analysis

The sigfit v1.1.0 R package (Gori and Baez-Ortega, 2018) was used to identify mutational processes (Alexandrov and Stratton, 2014), by fitting the mutational signatures published in the COSMIC catalog^2^ to the mutational profiles of the somatic SNVs in each tumor. The latter were obtained by classifying SNVs into 96 categories according to substitution type (interpreting the pyrimidine base in the Watson–Crick pair as the reference base) and the bases immediately 5′ and 3′ to the mutated base in the reference genome (Alexandrov and Stratton, 2014). Fitting of mutational signatures to somatic variants was initially performed using all 30 COSMIC signatures; subsequently, those signatures displaying significant activity and biological coherence were fitted again to obtain more precise estimates of signature activities.

### Gene Set Enrichment Analysis

The mutational landscape of ccRCC was explored through gene set enrichment analysis (GSEA), which was performed on those genes with *p* < 0.05 in the Fisher’s exact test of mutation burden (described above). This was performed via the Enrichr tool (Chen et al., 2013; Kuleshov et al., 2016) focusing on disease links through the Jensen Diseases database, which compiles evidence of gene–disease associations through the analysis of existing literature on genetic studies. A sensitivity analysis was performed at this stage by subsampling 71,000 random subsets of variable size (30–100 genes) from the prioritized gene set that were then evaluated in EnrichR, recording the proportion of times the terms were significant in the adjusted tests.

### Validation of Results

In order to validate our results, we accessed The Cancer Genome Atlas Kidney Renal Clear Cell Carcinoma (TCGA-KIRC) project data. To avoid potential confounders due to differences in the biological factors underlying cancer, patient exposures, and survival among people of different racial and ethnic backgrounds, we focused on the 417 samples with WES data available from white-ethnicity patients. For each prioritized gene in the mutational burden analysis, we recorded the number of somatic variants in deceased and alive patients in TCGA-KIRC. For that, we selected “kidney” in “Cases by Major Primary Site” classification available in the main page of TCGA portal. In filtering sets, we select “TCGA” program and TCGA-KIRC project. Results were subsequently filtered by “white” ethnicity. Each of the prioritized genes was manually entered to annotate the total number of patients with data for each gene and how many patients were deceased or alive. These data were subsequently analyzed via Fisher’s exact test as described above.

### Availability of Analysis Scripts and Data

A file with the putative somatic variation across all patients and the scripts used for the analyses in this study can be found in the GitHub repository^3^.

## Results

### DNA Extraction and Sequencing

We extracted and quantified genetic material from the original 13 patient FFPE samples for further evaluation via qPCR amplification with TaqMan probes of the housekeeping gene RNAsaP. Three of the samples were discarded from the study due to insufficient amount of extracted DNA and high fragmentation levels, caused by the formalin fixation process. We subsequently sequenced 20 paired DNA samples, extracted from normal and tumor tissues from the remaining 10 patients. The average age of the sequenced individuals was 55 years (range 31–80 years), where 70% were male and 40% died during the course of the study. Amplicon size ranged between 157 and 182 base pairs (bp), with a mean insert length of 172 bp. The Ion AmpliSeq^TM^ Exome RDY Kit yielded a median of 91.17% reads covering the on-target region with at least 20× depth. Sequencing metric summaries are shown in Supplementary Table S1.

### Variant Calling and Annotation

**FIGURE 1:**
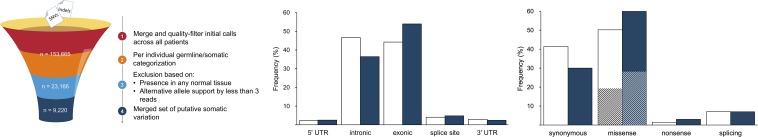
Summary of major variant call filtering steps (left panel) and annotation results identified in gene elements (middle panel) and of coding consequences (right panel; deleteriousness shown as striped sections). The reference dataset of germline variation is shown in white and the somatic variation is represented in blue.

A total of 122,019 SNVs and 31,646 indels were initially identified by the variant calling pipeline (Figure 1). The elevated number of indels was likely due to characteristic sequencing errors at polynucleotide tracts, associated with the Ion Torrent sequencing chemistry (Fujita et al., 2017; Lata et al., 2018). The categorization of all these variants into germline and somatic sets per individual and a filtering of the flagged variants resulted in a total of 23,157 SNVs (18.98%) and 9 indels (0.28%) of somatic origin. A final filter based on their presence in normal tissues from other patients resulted in a refined set of 9,220 (40%) high-confidence somatic SNVs, which were considered for subsequent analyses; all indels were filtered out at this stage. This figure agrees with previous results (Miao et al., 2018), confirming that ccRCC is among the cancer types with lowest somatic mutation prevalence. To provide further support to the putative somatic calls, we aligned the 9,220 somatic variants to that of COSMIC. In agreement with other studies, we found that 1,012 (10.9%) variants were represented in COSMIC (Cai et al., 2016), supporting their credibility. Besides, to evidence other footprints of somatic mutations, we predicted their functional consequences. As expected, comparing the somatic variation to a suitable reference germline variation set obtained from ExAC (Figure 1), we found somatic variants to be more prevalent than germline variants in exonic regions (54.0% vs. 44.3% of germline). Furthermore, somatic variation involved a larger proportion of missense (60.0% vs. 50.0% of germline) and nonsense (3.0% vs. 1.0% of germline) substitutions. Among missense substitutions, the somatic set also displayed a larger proportion of predicted deleterious mutations, on the basis of SIFT score (54.0% vs. 40.0% of germline missense).

Finally, to evaluate the evidence for selection on somatic substitutions and identify any potential contamination from germline polymorphisms, the ratio of nonsynonymous substitutions per synonymous site (dN/dS) was measured for the set of somatic variants using a dN/dS model optimized for the analysis of selection in cancer (Martincorena et al., 2017). This algorithm accounts for variation in mutation rates, sequence context and trinucleotide mutability to reliably estimate dN/dS ratios, providing exome-wide and gene-wise estimates of dN/dS ratios and significant departures from neutrality (dN/dS = 1). Somatic variants identified in more than one tumor (*n* = 464) were excluded from the analysis in order to avoid spurious inflation of dN/dS estimates. The analysis yielded an exome-wide dN/dS≈1, which is indicative of largely neutral evolution, in agreement with previous studies of selection in cancer (Martincorena et al., 2017). Similarly, none of the genes were found to display detectable evidence of selection on missense or truncating substitutions.

### Gene-Based Mutation Burden and Mortality by ccRCC

**FIGURE 2:**
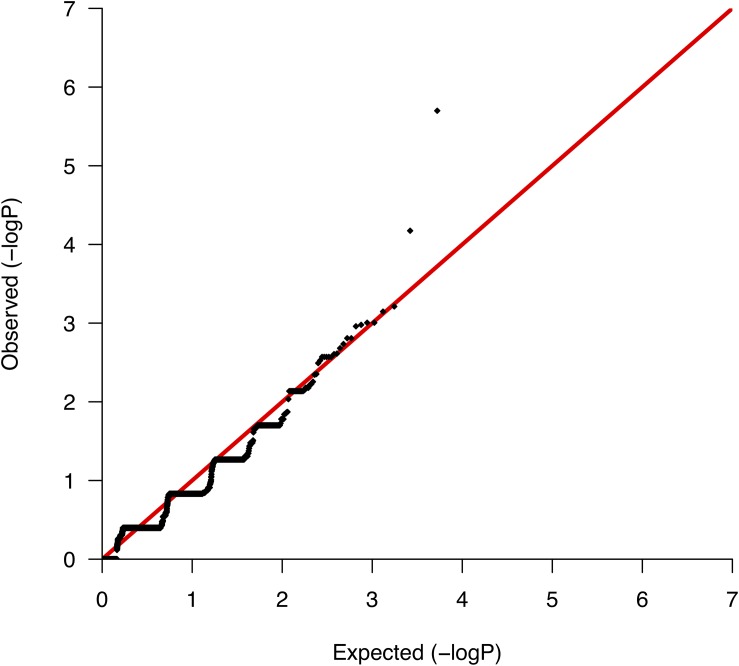
Quantile-quantile plot of the mutation burden test results from the association with mortality.

We conducted comparative analyses between surviving and nonsurviving ccRCC patients, testing for differences per gene in the somatic mutation burden. We found a total of 5,267 genes with evidence of somatic variation among the 10 patients, where the most altered gene in terms of the number of mutations was *CDC27*, which harbored a total of 89 somatic variants. We then applied Fisher’s exact test to rank the differences in the somatic burden and prioritized 138 genes based on nominal significance (lowest nominal *p* = 2.0 × 10^−6^; Supplementary Table S2). A QQ-plot of the distribution of gene-based *p*-values nearly followed the null (Figure 2) suggesting a minimal lambda factor (1.071) and a minimal inflation of results. Interestingly, among the genes ranking higher, we found a number of genes expressed in kidney tissues and previously associated with a variety of human malignancies of neoplastic and nonneoplastic origin, such as *GPR155* (ranked 1st), *INPP5K* (ranked 3rd), and *KRT7* (ranked 4th) (Supplementary Table S2). Another notable result was the presence in the list of various mucin-encoding genes (*MUC5B*, *MUC12*, and *MUC16*), which are commonly observed mutated in many cancers and have been previously linked to colorectal, ovarian and hepatological cancers (Yin et al., 2013; Felder et al., 2014; Wang et al., 2018), as well as to severe fibrotic lung disorders (Seibold et al., 2011). In agreement with a previous targeted sequencing study (Tennenbaum et al., 2017), the mutation burden of *VHL*, which is the main hallmark of ccRCC, showed no differences between survivors and nonsurvivors, supporting its role only during early stages of tumorigenesis (Mandriota et al., 2002; Mitchell et al., 2018).

### Gene Set Enrichment Analysis and Mutational Signatures

**FIGURE 3:**
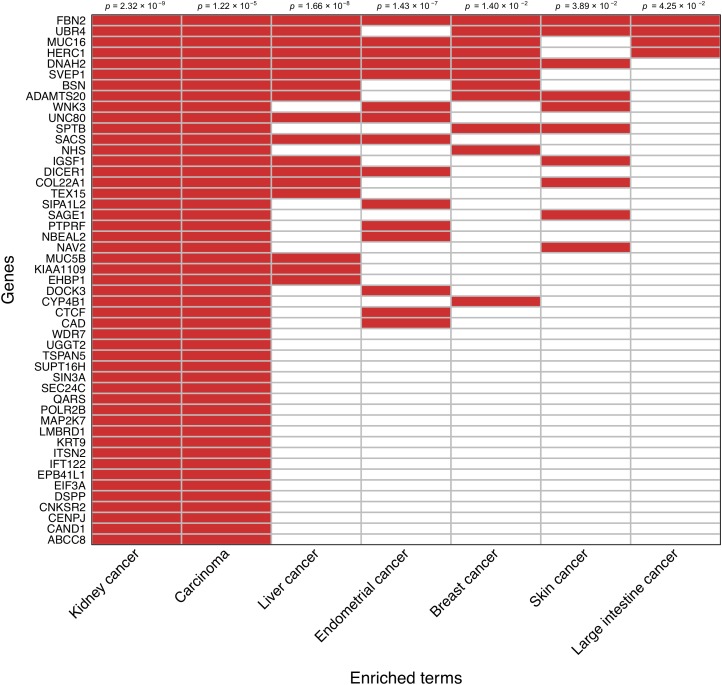
Clustergram representing the association of the subset of 49 prioritized genes that have direct links with kidney cancer. Relationships with other cancer types are also shown. Significance values are shown on the top.

An enrichment analysis focused on the set of 138 prioritized genes based on the somatic mutation burden differences between survivors and nonsurvivors was performed to reveal disease links according to the Jensen Diseases database. Those genes most likely to be driving such relationships were prioritized. In agreement with a visual inspection of the prioritized gene list, this analysis showed a strong association between these genes and both kidney cancer development (adjusted *p* = 2.32 × 10^−9^) and carcinoma development (adjusted *p* = 1.22 × 10^−5^). A random subsampling of 71,000 gene sets of variable size out of the 138 prioritized genes also provided significant support to the enrichment of these two terms 90.9 and 74.5% of the times, respectively. This also evidenced that as few as 20% of randomly sampled genes from the prioritized gene set still detected a significant enrichment of kidney cancer 60% of the times, and that such statistical support stabilized when considering a random sample of 50% of the prioritized genes (Supplementary Figure S2). This sensitivity analysis reinforced that these observations are not significant by chance. A clustergram of the 49 genes that were directly associated with kidney cancer development is shown in Figure 3.

**FIGURE 4:**
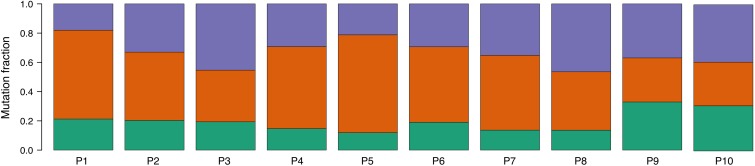
Proportion of COSMIC signatures displaying significant activity in each patient tumor. Color code correspondence is: green, signature 1; red, signature 5; and purple, signature 12.

An analysis of mutational signatures revealed three signatures (COSMIC signatures 1, 5, and 12) with significant activity in the tumors (Figure 4). Signatures 1 and 5 correspond to endogenous mutational processes that are consistently operative in nearly all human cells (Alexandrov et al., 2015). On the other hand, signature 12, whose etiology is presently unknown, has been previously described only in liver cancer, and thus its presence in ccRCC tumors is unprecedented (Alexandrov et al., 2013). Strikingly, although with borderline significance, the mutational contribution of signature 12 in the tumors tends to associate with the age at diagnosis (rho = 0.71, *p* = 0.02). Notwithstanding this result, the overall somatic mutation burden was not correlated with the age at diagnosis (rho = 0.32, *p* = 0.36).

### Validation of Results

In order to provide validation to our findings, we then retrieved the mutation count for all of the prioritized genes from the TCGA-KIRC dataset, stratified by survival, and again tested for differences per gene in the somatic mutation burden. Only two out of the 138 prioritized genes were nominally significant in the TCGA-KIRC dataset, namely *SIPA1L2* (*p* = 0.036), which encodes the signal-induced proliferation-associated 1 like 2, and *EIF3A* (*p* = 0.048), which encodes the eukaryotic translation initiation factor 3 subunit A. Both of these genes have been previously linked to unfavorable prognosis in other cancers (Minato and Hattori, 2009; Yin et al., 2018).

## Discussion

Despite previous attempts to reveal molecular prognostic signatures of ccRCC based on multiomics data (The Cancer Genome Atlas Research Network, 2013), this study constitutes the first exome-wide approach for revealing genes with differential accumulation of somatic mutations in relation to cancer-associated mortality in ccRCC patients. Previous studies assessing links with prognosis or responses have either used targeted approaches directed to a limited set of genes that commonly accumulate mutations (Tennenbaum et al., 2017) or to evidence associations with treatment responses to the therapies (Fay et al., 2016; Miao et al., 2018). At most, these studies revealed that recurrent mutations in *PBRM1*, one of the well-known ccRCC genes, might have implications in the treatment responses. In contrast, our study enabled prioritizing 138 genes based on a refined set of putative somatic SNVs, where 49 of those genes had previously been related to kidney cancer according to the literature, and two genes (*SIPA1L2* and *EIF3A*) were validated in independent datasets from TCGA. Our study has also yielded evidence suggestive of the activity of COSMIC signature 12 in kidney cancer, in addition to two well-established endogenous mutational processes.

*SIPA1L2* is a renal cancer biomarker according to The Human Protein Atlas^4^, as its expression associates with a significant unfavorable prognosis. Members of this family encode mitogen activators of GTPase activity for Ras-related Rap regulatory proteins, contributing as essential regulators in cell cycle and of metastasis in various types of solid cancers. Overexpression of its mouse ortholog *in vitro* alters primary mammary tumor cell morphology and adhesive properties (Zhang Y. et al., 2015), inducing detachment of cells from the matrix, while it increases the metastatic capacity *in vivo* (Park et al., 2005). Consistently, primary tumors with metastasis express more protein that nonmetastatic tumors for a variety of solid tumors (Park et al., 2005; Minato and Hattori, 2009). Therefore, its role in ccRCC prognosis may well be linked to this influential activity in cancer invasiveness. With respect to *EIF3A*, it encodes the largest subunit of eIF3 complex involved in translational regulation, cell growth and cancer. The eIF3a subunit itself is suggested to regulate a subset of mRNAs including factors required for development and differentiation. Its expression is ubiquitous, although at higher levels in adult proliferating tissues and in a number of cancer types, suggesting that it is required for cell proliferation and that is a negative regulator of cell differentiation (Saletta et al., 2010). In fact, in some cancer types, there is a correlation between higher eIF3a expression and the metastatic ability (Bachmann et al., 1997). The expression of some of the eIF3 subunits, including eIF3a, have been associated with cancer prognosis and therapeutic response (Saramäki et al., 2001; Buttitta et al., 2005; Yin et al., 2018). Interestingly, eIF3a was also involved in fibrosis in humans, particularly in renal fibrotic tissue, by regulating the TGF-β1/SMAD3 signaling pathway (Zhang Y.-F. et al., 2015). Based on this evidence, eIF3a may be associated with ccRCC prognosis by its central role in the maintenance of the malignant status of cells.

Despite we were unable to validate their association with ccRCC mortality, there were other prioritized genes among the top ranked that are interesting candidates for further study, namely *GPR155*, *INPP5K*, *KRT7*, *CYP4B1* and the mucin encoding genes (*MUC5B*, *MUC12*, and *MUC16*). Most of these have been associated with tissue remodeling and cancer processes (Imaoka et al., 2000; Hedberg Oldfors et al., 2015; Giunchi et al., 2016; Umeda et al., 2017; Renshaw and Gould, 2018; Sarlos et al., 2018). Interestingly, albeit previous studies have argued that some of the MUCs should be excluded as cancer genes based on biological relevance (Lawrence et al., 2013), there is much evidence supporting the link between *MUC12* and *MUC16* and cancer development and evolution (Matsuyama et al., 2010; Yin et al., 2013; Felder et al., 2014; Wang et al., 2018). The results for *MUC5B* are less clear, since there is not a direct evidence in the literature of a link with oncogenic processes, but only with susceptibility and survival in pulmonary fibrosis through germline regulatory variants (Seibold et al., 2011; Fingerlin et al., 2013; Noth et al., 2013; Peljto et al., 2013; Allen et al., 2017). Curiously, a recent WES study by Lata et al. (2018), aimed at providing diagnosis of adult probands with CKD of unknown cause, identified causal germline mutations in *PARN* [poly(A)-specific ribonuclease], another pulmonary fibrosis susceptibility gene (Stuart et al., 2015). In agreement with these results, some studies have argued in support of pathogenic similarities between pulmonary fibrosis and cancer (Vancheri, 2015). Under such scenario, it could be speculated that coding mutations in *MUC5B* and *PARN* may play a role in oncogenesis in lung and kidney tissues.

One of the most notable strengths of this study is that it focuses on a homogeneous patient population, with all patients being included at stage IV and, similarly treated. Besides, the combination of high-depth WES of matched tumor–normal sample pairs from each patient, and the multiple filtering routines performed after variant calling, enabled the derivation of a high-confidence set of somatic variants. This led to findings which, for two of the resulting prioritized genes, were validated in an independent dataset from a much larger ccRCC patient population. The limited number of genes that validate in TCGA-KIRC can be explained by the limited sample size analyzed in this study, and by the systematic analytic differences between the two studies. Notwithstanding, we also acknowledge some salient limitations in the study. First, the study evaluated a very small patient sample and focused on the high-mortality risk spectrum of ccRCC cases that may not be representative of the full disease spectrum. Despite the similarity in demographic and clinical data of patients irrespective of the mortality, the limited sample size precluded a formal survival analysis accounting for time-dependent risks while adjusting for potential confounders. Therefore, the results should be taken with caution, as there may be alternative patient variables that can explain the associations found. Second, we only sequenced a single specimen at the point of patient diagnosis and did not analyze WES of metastasis in remote tissues. As a consequence, our capacity to identify candidate genes linked to ccRCC survival was limited to those at the pre-treatment stage, hindering the possibility of identifying additional genes as the tumors responded to therapy or the effect of mutations in metastases. Third, because of the capture design of WES, we were unable to assess the association of noncoding variants with patient mortality, as has been previously suggested for regulatory variants in the telomerase reverse transcriptase encoding gene *TERT* (Casuscelli et al., 2017). Fourth, structural variants are common in ccRCC (Thiesen et al., 2017) and have been associated with poorer prognosis (Chen et al., 2009; Moore et al., 2012). However, we did not explore the implications of these on patient mortality because we anticipated that they would be highly uncertain in our data. The reasons for this are: (i) the algorithms used for calling SNVs and small indels are not well suited for the detection structural variation, which require dedicated algorithms; (ii) WES has limited sensibility for the detection of structural variants; (iii) there is an extensive lack of agreement between the structural variant callers, implying that their calls necessitate a consensus of multiple algorithms; and (iv) we lacked WES data from a pool of references to serve as controls for the AmpliSeq technology to be used for calling structural variants. Finally, we did not adjust for multiple hypothesis testing because our analyses were exploratory in nature: our interest lay in maximizing the utility of the available data for this small patient sample, to identify a sufficient number of prioritized genes as to power our gene set enrichment analysis. Hence, we tolerated the existence of false positives as a reasonable trade-off for an enhanced gene prioritization, which was necessary for the enrichment analysis. As such, our results should be regarded as hypothesis-generating findings.

## Conclusion

In this study, we identified and validated two genes that are recurrently altered in ccRCC tumors and that associate with patient mortality. These have been previously suggested as biomarkers of cancer prognosis and participate in molecular pathways linked to tumor development and progression. Additionally, we provide evidence suggestive of the activity of COSMIC mutational signature 12 in kidney cancer, hinting at a potentially incomplete understanding of the mutational processes that are involved in this kind of malignancy. Independent validation studies achieving larger statistical power are needed to better evaluate the impact on ccRCC patient mortality.

## Ethics Statement

This study was carried out in accordance with the recommendations of the Ethics Committee for Clinical Research from the Hospital Universitario Nuestra Señora de Candelaria in accordance with the Declaration of Helsinki. The protocol was approved by the Ethics Committee for Clinical Research from the Hospital Universitario Nuestra Señora de Candelaria.

## Author Contributions

AB-O, AM-A, BG-G, and CF wrote the manuscript and the Supplementary Files. BG-G and SL-L performed DNA extractions and quantifications, as well as the preparation of samples for sequencing. CH-P, M-d-CM, and MM performed data collection and the record of patient and sample features. AB-O and JL-S conceived and implemented software procedures. AM-A, CF, and JL-S performed the statistical tests on detected somatic variants. CF provided a general supervision of the project, giving guidelines for each step. All the authors provided insights, corrections and approved the final version of the manuscript.

## Conflict of Interest Statement

The authors declare that the research was conducted in the absence of any commercial or financial relationships that could be construed as a potential conflict of interest.

## Footnotes

1 https://cancer.sanger.ac.uk/cosmic/

2 https://cancer.sanger.ac.uk/cosmic/signatures

3 https://github.com/genomicsITER/mutational-burden-and-ccRCC-mortality

4 https://www.proteinatlas.org/ENSG00000116991-SIPA1L2/pathology

## Supplementary Material

FIGURE S1 Overview of the variant calling pipeline. Aligned BAM files are input to generate a final set of genomic variants for subsequent analysis.

FIGURE S2 Proportion of significant results in the gene set enrichment analysis for “kidney cancer” and “carcinoma” as a result of the random sampling of gene sets from the prioritized gene set.

TABLE S1 Whole-exome sequencing summary metrics for each sample. Average for both normal and tumor samples is shown (minimum and maximum values in parenthesis).

TABLE S2 Ranking of prioritized genes sorted by significance level.
