# Supplementary figures and images for: Whole-Exome Sequencing Identifies Somatic Mutations Associated With Mortality in Metastatic Clear Cell Kidney Carcinoma
Source: Front Genet. 2019 May 15;10:439. doi: 10.3389/fgene.2019.00439 (PMC6529576; doi:10.3389/fgene.2019.00439)

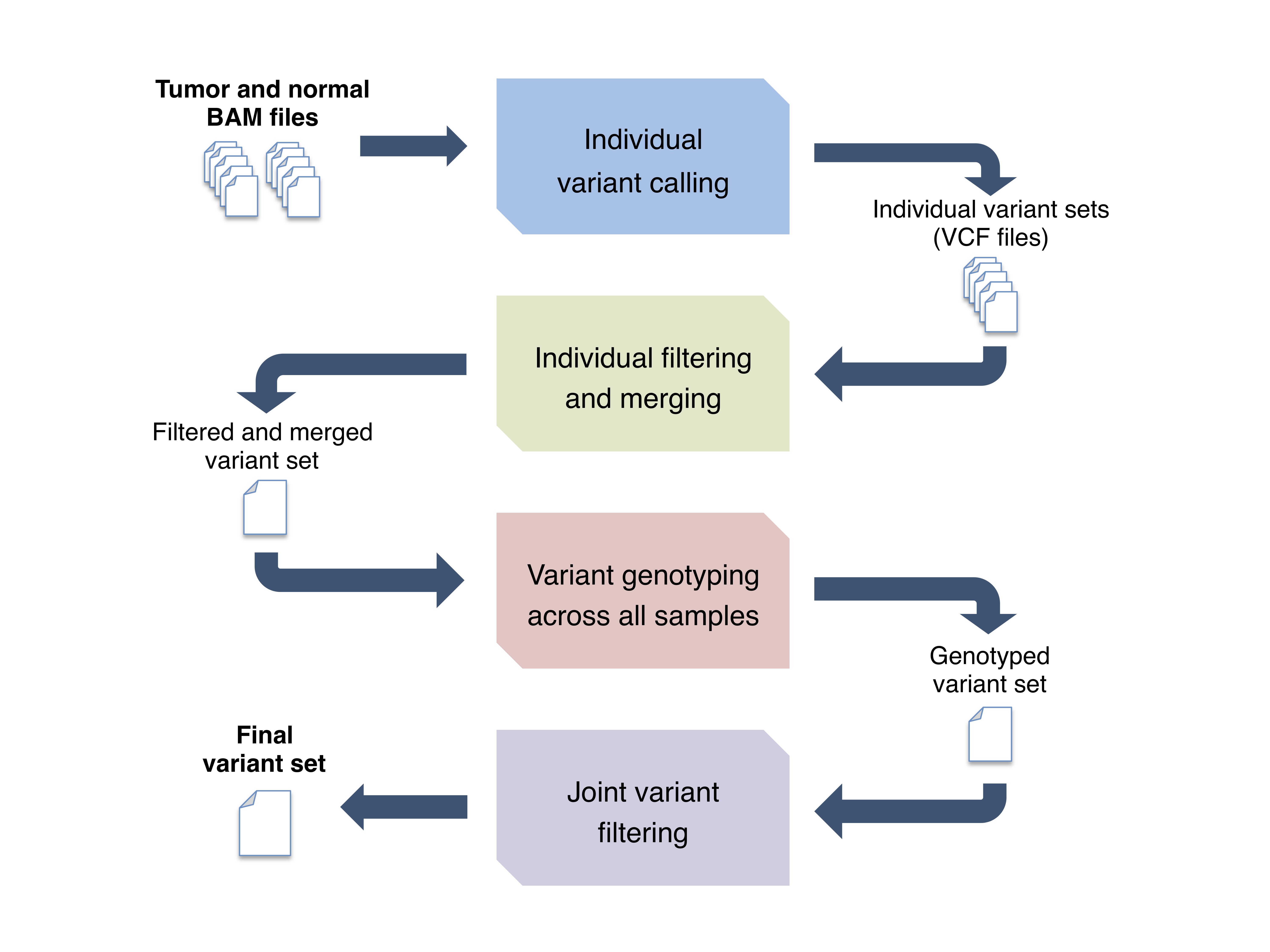

Supplement: FIGURE S1 — Overview of the variant calling pipeline. Aligned BAM files are input to generate a final set of genomic variants for subsequent analysis. [file Image_1.TIFF]

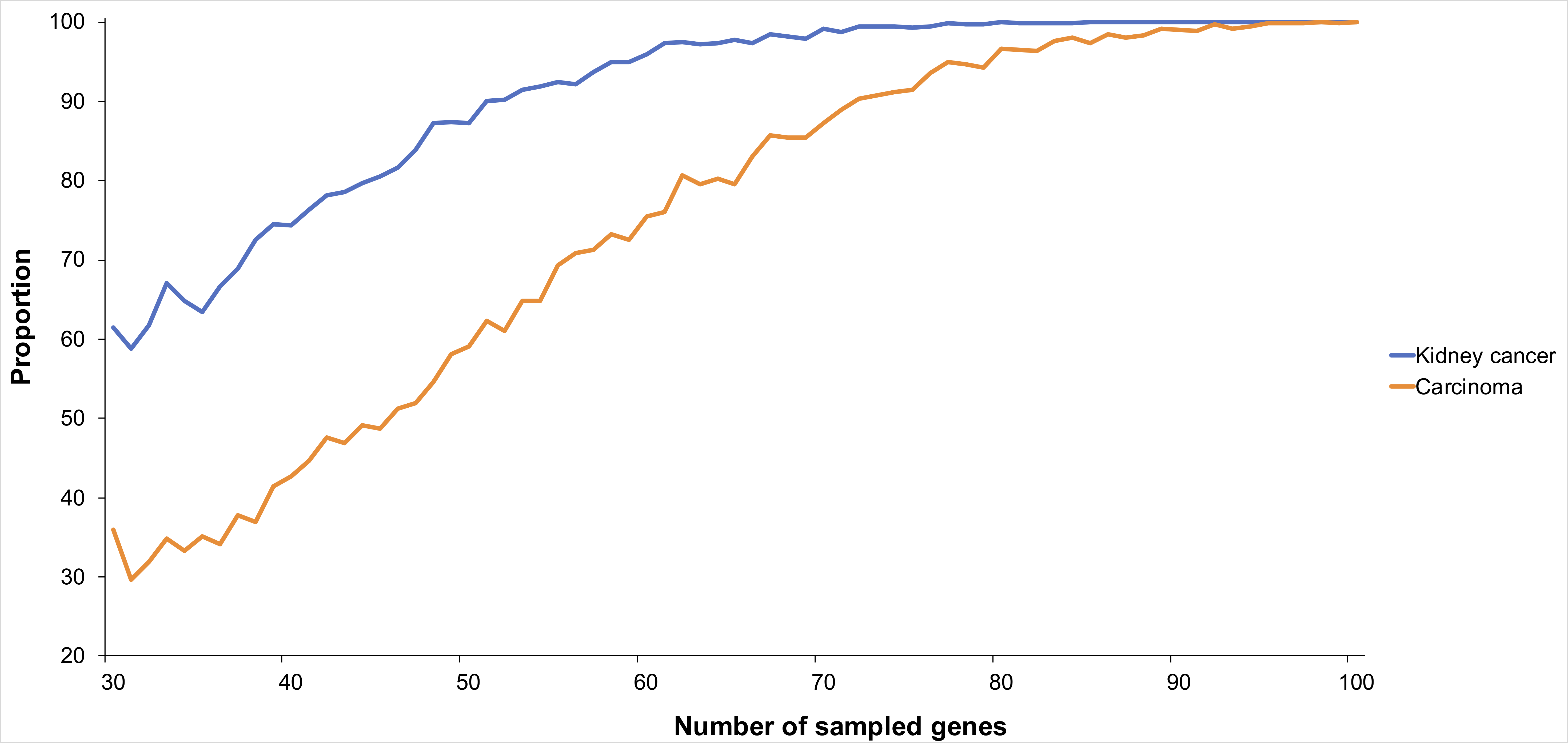

Supplement: FIGURE S2 — Proportion of significant results in the gene set enrichment analysis for “kidney cancer” and “carcinoma” as a result of the random sampling of gene sets from the prioritized gene set. [file Image_2.TIFF]
